# Awareness of atrial fibrogenesis and natriuretic peptide release

**DOI:** 10.1007/s12471-017-0955-6

**Published:** 2017-02-09

**Authors:** W. C. F. W. Meijers, T. Jaarsma, D. J. van Veldhuisen, T. Hoekstra, R. A. de Boer

**Affiliations:** 1 Department of Cardiology, University Medical Centre, University of Groningen, Groningen, The Netherlands; 2 Faculty of Health Sciences, Linköping University, Linköping, Sweden

We thank dr. Lucas for her comments regarding our article in which we described patients with heart failure with preserved ejection fraction (HFpEF) and low levels of natriuretic peptides. In this article [1], we described that HFpEF patients with relatively low B‑type natriuretic peptide (BNP) levels have strikingly similar clinical characteristics as HFpEF patients with elevated BNP levels, except for BMI, which was significantly higher in the first group. Clinicians might expect that low BNP levels are associated with low risk of adverse events, but, as recently observed in another study [2], NP-proBNP and BNP are not the ideal markers to identify low-risk patients.

The hypothesis, shared by dr. Lucas, that the stiff left atrial syndrome might explain the low BNP levels observed in HFpEF patients is interesting. The stiff left atrium has mostly been described after ablation therapy for atrial fibrillation (AF) [3, 4]. However, we agree that also in heart failure, atrial tissue may remodel and become stiff. It has indeed been suggested that heart failure patients with a longer duration of AF exhibit advanced remodelling of the atrial tissue, in which atrial cardiomyocytes are replaced by fibrotic tissue, a hallmark of remodelling [5]. The remaining atrial tissue no longer produces natriuretic peptides, with pseudo-low natriuretic peptide levels as a result [6].

However, in our study we observed a comparable prevalence of AF in both groups (BNP <100 pg/mL vs. BNP >100 pg/mL, 50% AF in both groups), suggesting the severity of atrial remodelling is equal. Further, advanced atrial remodelling would most likely be accompanied by higher levels of inflammatory and fibrotic biomarkers. But the levels of galectin-3, a fibrotic protein associated with worse outcome [7], and interleukin-6, an inflammatory biomarker, were comparable between both groups.

Preferably, we would have assessed cardiac and atrial dimensions by echocardiography, but, unfortunately, we do not have these data in the COACH database and acknowledge this as a limitation of our study.

So, based on the data presented, we cannot demonstrate evidence that the low BNP levels in our study were due to progressed atrial remodelling. But we agree that studying the left atrium is of importance in HF patients with paroxysmal or persistent AF and in patients who have pseudo-low BNP levels. Indeed, stiffening of the left atrium may clinically be under-recognized and more awareness is needed.
